# RNA metabolism is the primary target of formamide *in vivo*

**DOI:** 10.1038/s41598-017-16291-8

**Published:** 2017-11-21

**Authors:** Rafael Hoyos-Manchado, Félix Reyes-Martín, Charalampos Rallis, Enrique Gamero-Estévez, Pablo Rodríguez-Gómez, Juan Quintero-Blanco, Jürg Bähler, Juan Jiménez, Víctor A. Tallada

**Affiliations:** 1 Centro Andaluz de Biología del Desarrollo, Universidad Pablo de Olavide/Consejo Superior de Investigaciones Científicas, Carretera de Utrera Km1, 41013 Seville, Spain; 2 Research Department of Genetics, Evolution and Environment and UCL Institute of Healthy Ageing, University College London, WC1E 6BT, London, United Kingdom; 3 School of Health, Sport and Biosciences, University of East London, E15 4LZ London, United Kingdom; 4 Department of Human Genetics, McGill University, Montreal, H3A 0C7 Quebec, Canada; 5 Human Brain Mapping Unit, Instituto Pluridisciplinar, Universidad Complutense de Madrid, 28040 Madrid, Spain

## Abstract

The synthesis, processing and function of coding and non-coding RNA molecules and their interacting proteins has been the focus of a great deal of research that has boosted our understanding of key molecular pathways that underlie higher order events such as cell cycle control, development, innate immune response and the occurrence of genetic diseases. In this study, we have found that formamide preferentially weakens RNA related processes *in vivo*. Using a non-essential *Schizosaccharomyces pombe* gene deletion collection, we identify deleted loci that make cells sensitive to formamide. Sensitive deletions are significantly enriched in genes involved in RNA metabolism. Accordingly, we find that previously known temperature-sensitive splicing mutants become lethal in the presence of the drug under permissive temperature. Furthermore, in a wild type background, splicing efficiency is decreased and R-loop formation is increased in the presence of formamide. In addition, we have also isolated 35 formamide-sensitive mutants, many of which display remarkable morphology and cell cycle defects potentially unveiling new players in the regulation of these processes. We conclude that formamide preferentially targets RNA related processes *in vivo*, probably by relaxing RNA secondary structures and/or RNA-protein interactions, and can be used as an effective tool to characterize these processes.

## Introduction

Conditional mutations can be used as a “genetic switch” to study essential genes since they allow the propagation and genetic crosses under a permissive condition but also the study of loss-of-function under a restrictive condition. In yeast, temperature has been the most prominent condition used to facilitate this switching^1^. This ability to switch has enabled the identification of hundreds of mutant genes important for many biological functions, and revealed unexpected connections between pathways. However, not all proteins or positions within the primary sequence are sensitive to temperature changes^2^ and some genetic pathways are less prone to temperature-sensitive mutations^3^.

Thus, the use of other conditions such as chemical sensitivity that either target a given genetic pathway, a specific kind of molecules, loosen physical interactions in general terms or act as a non-functional analogue, greatly extends the number of conditional mutations in essential genes to be studied in order to draw better pictures of biological processes. Examples of those include heavy water^4^, ethanol^5,6^, glycerol and sorbitol^7^, caffeine^8,9^, camptothecin^10^, hydroxyurea^11^, ATP analogues^12^, cycloheximide^13^, DMSO^14,15^, or TBZ^16^. New sensitive alleles may also provide additional advantages, such as when studying complex genetic interactions. In such studies, it is desirable to have different switches to turn off one or more functions at the same time. Furthermore, these switches do not expose cells to heat-shock-derived stress, do not need complex equipment to control temperature when studying living cells under the microscope and enable quick reversibility studies (adding a drug to and washing a drug out of a well^17^, is much faster than heating up and cooling down the whole optical setup).

The fission yeast *Schizosaccharomyces pombe* is a powerful genetic model, with the major signalling pathways and cellular processes remaining conserved between yeasts and mammals. Genome-wide non-essential gene deletions studies and extensive screens using conditional mutations have been carried out successfully in this model and have historically boosted our knowledge of gene function, especially in cell cycle regulation^18^. However, there are still a number of key elements, fine tuning regulators and genetic pathways connectors that remain unknown. In this work, we have combined these two approaches to search for formamide hyper-sensitive deletions and mutations in fission yeast, with the aim of uncovering both the *in vivo* effect of this molecule and identifying new genes involved in cell cycle regulation. Formamide (HCONH_2_) is an amide derived from formic acid which acts *in vitro* as an ionic solvent that destabilizes non-covalent bonds^19,20^. It is traditionally used in protocols such as Fluorescence *in Situ* Hybridization (FISH) or cell-free *in vitro* translation where it is desirable to relax mRNA secondary structures. However, there is very little information about its molecular mode of action in living cells. Formamide is a small molecule that can easily enter the cell and, importantly, is not metabolized. It has also been shown to lack mutagenic properties^21^ and formamide sensitive mutants were previously reported in budding yeast^22^. All these properties make formamide a good candidate for genetic screens.

## Results

### Formamide limits growth rate in fission yeast

**Figure 1:**
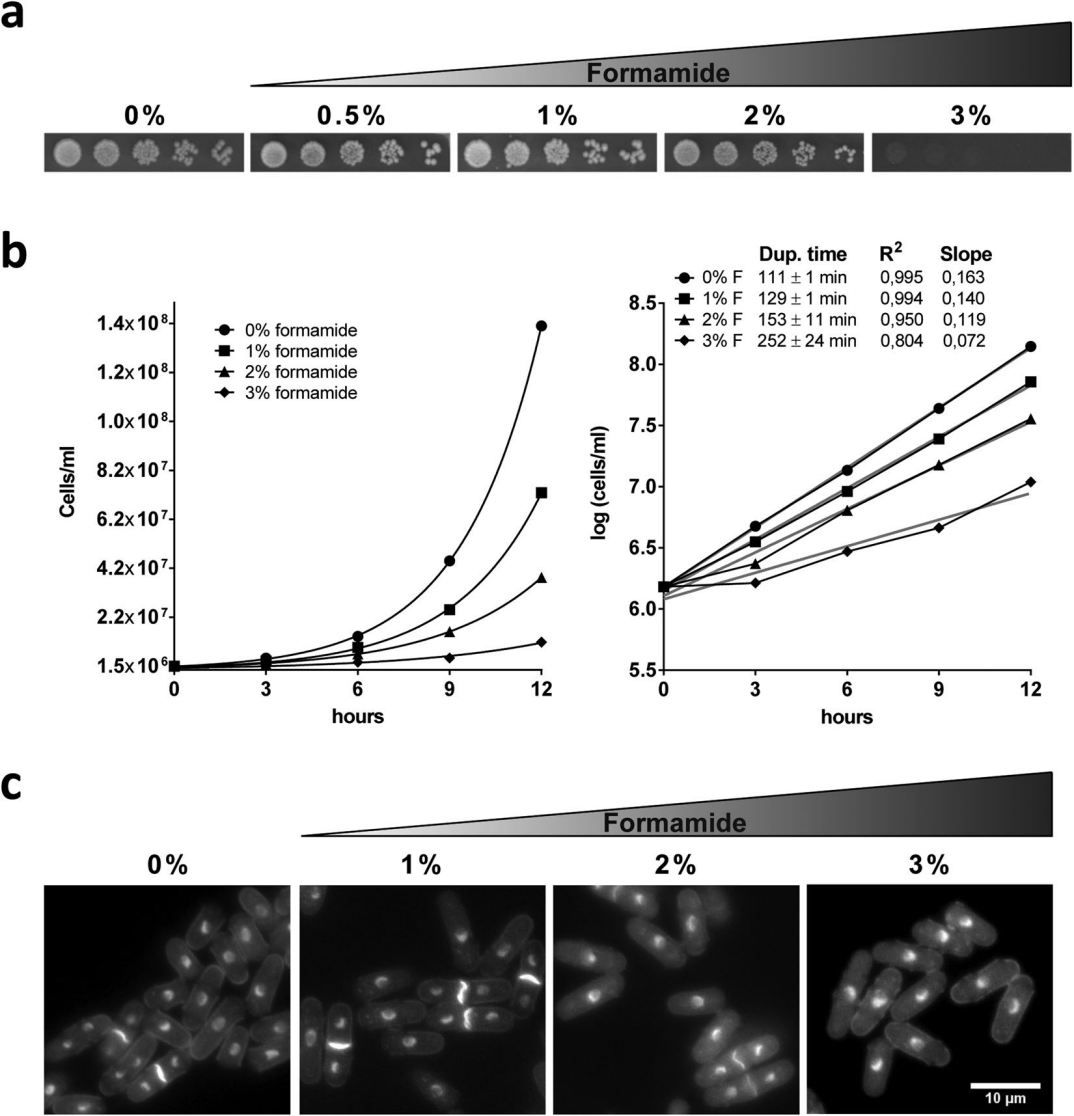
Limiting effect of formamide on fission yeast proliferation. (**a**) Five-fold serial dilutions were spotted on YES plates for 3 days at 30° in the presence of indicated formamide concentrations (v/v). (**b**) Growth curves of *S. pombe* in rich liquid media in the presence of 0%, 1%, 2% and 3% respectively. Normalized values by initial number of cells and averaged from two biological repeats are plotted (left). Duplication time in each concentration was calculated by interpolating from average logarithmic equations (right). Logarithmic lines in grey correlation coefficient and line slopes are also indicated. (**c**) DAPI/Calcofluor staining of cells incubated for 12 hours from a starting density of 1.5 × 10^6^ cells/ml at the respective concentrations of formamide. Cells in the absence of formamide reach stationary phase, becoming smaller as they starve; while in the presence of formamide over the same time, cell cultures do not reach stationary phase since growth rate is delayed. Scale bar: 10 µm.

To our knowledge, the only report studying fission yeast survival rates in the presence of different concentrations of formamide was provided by Abbondandolo *et al*.^23^. In this study, survival rates were estimated after incubation of cells up to 24 hours in buffer solution. Thus, cells were not actively dividing while in the presence of the drug. We aimed to determine the limiting and lethal concentrations of formamide for the fission yeast wild type (wt) strain during active growth in both solid and liquid media at the standard temperature of 30°. First, we plated wt cells in serial dilutions (1/5) on YES agar media containing 0%, 0,5%, 1%, 2% and 3% v/v formamide respectively. As shown in Fig. 1a, fission yeast is able to grow well up to 2%. However, in contrast to budding yeast^22^, *S. pombe* does not proliferate when grown on 3% formamide in solid media.

In order to address how the growth rate is affected in exponential cultures at reference concentrations (0%, 1%, 2% and 3%), we followed duplication time in liquid media by scoring the number of cells/ml every 3 hours for 12 hours in two independent biological repeats. We found that duplication time is longer in the presence of formamide in a concentration-dependent manner. The wild type population doubled every 111 ± 1 minutes in regular YES media at 30° while they took 129 ± 1, 153 ± 11 and 252 ± 24 minutes in the presence of 1%, 2% and 3% formamide respectively (Fig. 1b). As was observed using solid media, further incubation in liquid media allowed wild type cells to reach saturation at sub-lethal concentrations (1% and 2%). However, adding 3% v/v formamide resulted in a severe delay of growth rate during the same time frame and more importantly these cultures did not reach saturation. DAPI/Calcofluor co-staining of wt cells growing on either concentration during 12 hours did not show significant phenotypic differences in comparison to regular YES (Fig. 1c). Based on these results, we decided to use 2% v/v for fission yeast as the reference concentration in further experiments. It is worth noting that wild type cells are not able to proliferate when formamide is added to EMM solid media and do so very poorly in liquid even at lower concentrations. Ammonia and urea are used as an inorganic nitrogen source in yeasts and other organisms and formamide – an NH_4_^+^ analogue – seems to outcompete the metabolism of these compounds very efficiently^24–27^.

### Isolation of new conditional mutants in fission yeast

**Figure 2:**
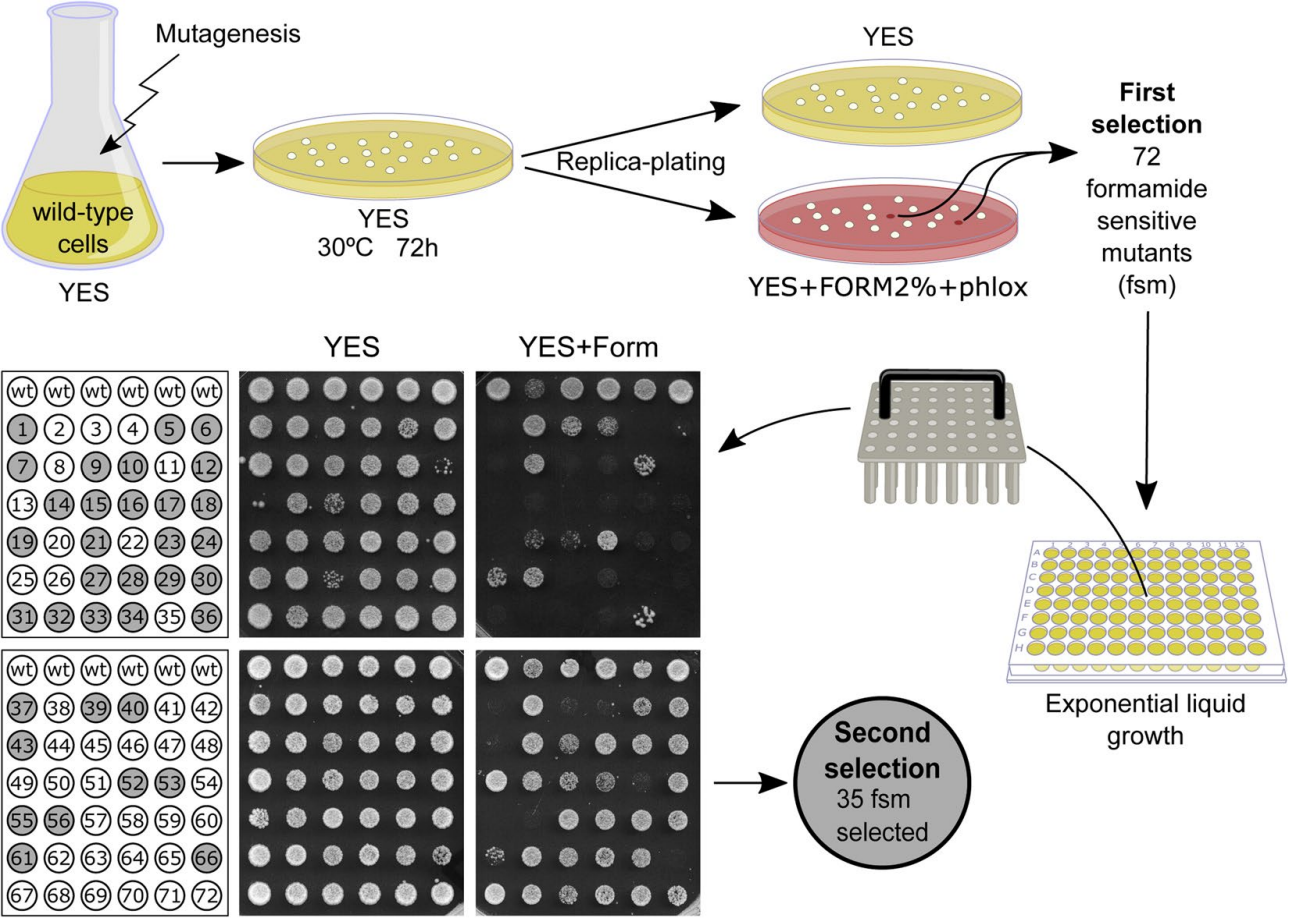
Formamide sensitive mutant screen design. Schematics of formamide sensitive mutations (*fsm*) searching strategy. Two selection rounds were performed in order to select the tightest alleles.

Previous work in budding yeast identified conditional mutants hyper-sensitive to the denaturing agent formamide^22^. From then on, a handful of existing mutations have demonstrated sensitivity to this drug, but always as a secondary phenotype^28–35^. Thus, there is very little information about the effect of this drug *in vivo*. Using the Ames test, it has been reported that formamide does not have mutagenic properties neither on its own nor after S9 liver homogenate treatment^21^. However, it is not known if sets of molecules or genetic pathways are especially sensitive or whether the drug simply lowers biochemical efficiency in general terms by relaxing weak non-covalent interactions. Formamide has never been used in fission yeast studies as a restrictive condition. Thus, by using this new condition we aimed to find new genes involved in cell cycle regulation that may have escaped identification in other screens. To this end, we mutagenized an exponentially growing culture in four different ways to widen the expected range of mutations (see Materials and Methods). After mutagenesis, a total of 60000 cells were plated in rich media (YES) and incubated for 4 days at 30°. Resulting colonies were replica-plated back onto YES and YESFPh (YES containing 2% formamide and 10 mg/L Phloxine B) to identify those who were able to grow normally in YES plates but died - or were very sick - in the presence of formamide (Fig. 2). A total of 72 Formamide Sensitive Mutants (named *fsm1* to 72) were initially isolated. To reduce the number of mutants and obtain a list of only the most formamide sensitive strains, the mutants were grown in regular rich liquid media until log phase and plated as a single spot in the presence of formamide. We narrowed down to 35 severely sensitive strains for further analysis (Fig. 2).

**Figure 3:**
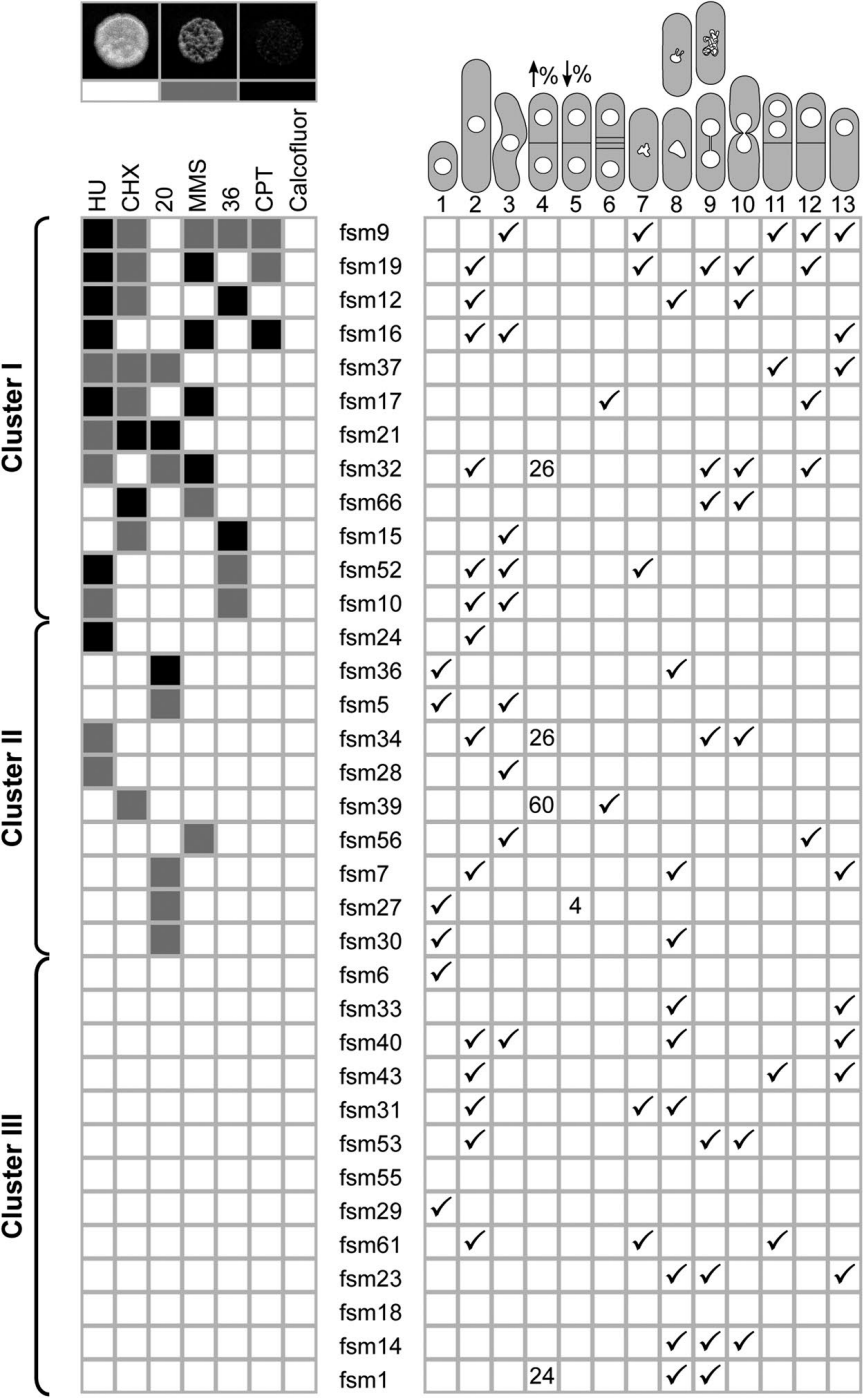
Global characterization of *fsm* mutants. Clustering of *fsm* alleles by other sensitivities to widespread conditions. A colour code was assigned for three degrees of sensitivity in comparison to wild type standards onto the same plate: Solid black for severe, light grey for intermediate and white for no sensitivity. An example of each is shown on top. *Fsm* strains can be grouped in three distinct clusters. About one third of formamide sensitive mutants (cluster III) show no sensitivity to other conditions tested. Right panel ticks most obvious morphology and cell cycle phenotypes for each mutant based on DAPI/calcofluor co-staining after 4 hours in the presence of formamide. Numbers in categories 4 and 5 represent septation percentage as compared to 8% found in wild type control and most of the mutants in the same condition. Representative phenotype cartoons are numbered on top to be referred to in Fig. 4 and Supplementary Figure 3.

First, we asked if the biological base of formamide sensitivity could be separated from other widespread restrictive conditions previously used in fission yeast by plating all mutants in camptothecin, hydroxyurea, calcofluor white, cycloheximide, methyl methanesulfonate (MMS), as well as low (20°) and high temperature (36°). *Fsm* mutants were sensitive to these conditions in different combinations and have different penetrance (Fig. 3). To cluster mutants according to their sensitivity to each condition, we assigned the following colour code: Solid black for lethal, grey for slow growers (in comparison to wild type controls on the same plate) and white for non-sensitive (Fig. 3 top left panels). This way, mutants were clustered by the number of sensitive conditions apart from formamide. As can be seen in Fig. 3 (left panel), we grouped all strains in three different clusters with about a third of strains in each: sensitive to several (two or more) conditions (cluster I), sensitive to one extra condition (cluster II) and exclusively sensitive to formamide (cluster III). The fact that we find such phenotypic polymorphism in response to different drugs and temperature in clusters I and II, suggests that different genetic pathways may somehow be affected in this batch of mutants. This could be explained either by the presence of multiple mutations or single mutations in genes involved in general processes that give rise to pleiotropic defects. To further investigate these possibilities, we crossed thirteen *fsm* mutants (three strains from cluster I; three from cluster II and seven from cluster III) to a wild type to analyse progeny. Tetrad dissection showed that only two mutations did not consistently segregate as monogenic (2:2) for formamide sensitivity. Eleven remaining strains segregated as expected for single locus mutations (Supplementary Figure 1). This suggests that most of the *fsm* mutants affected a single locus. Furthermore, formamide sensitive pairs in tetrads pulled from cluster I (*fsm*9, 16, 19 and 32) were also sensitive to the expected agents previously found for each mutant (see Fig. 3 and Supplementary Figure 2). This also suggests that the multiple sensitivity phenotype in these strains is due to a single-locus mutation rather than mutations in several loci.

In this screen, we also found 13 strains which, out of all conditions tested, were only sensitive to formamide (cluster III). Although other sensitivities could be tested, it is likely that at least some of these strains are exclusively sensitive to formamide. To check that mutations in this cluster were not in the same gene, we built complementation groups. To this end, 6 strains were crossed to each other and progeny were screened for wild type recombinants. Such recombinants were found in all cases; indicating that at least 6 mutations in cluster III correspond to as many independent loci.

**Figure 4:**
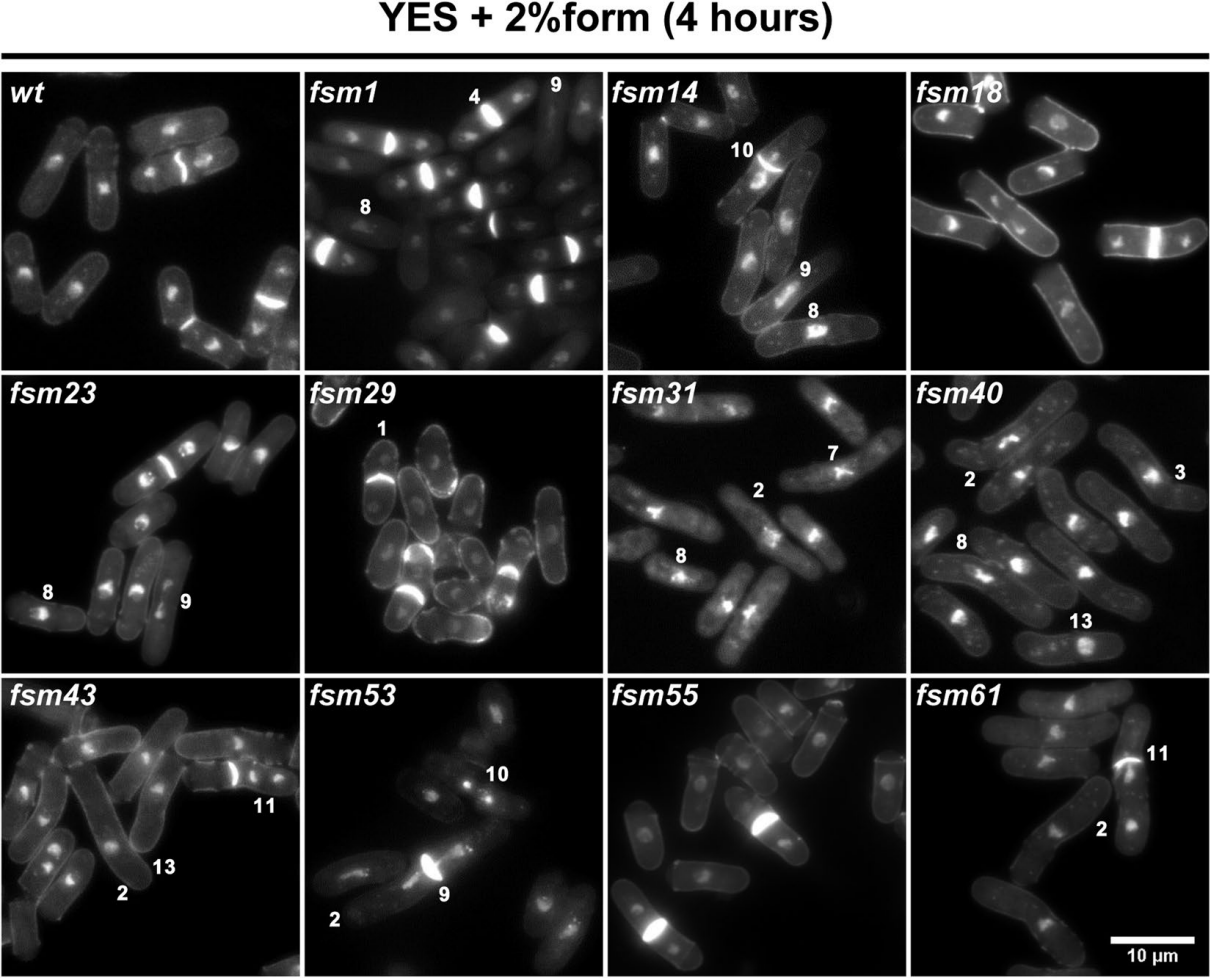
Phenotype range in *fsm* strains in cluster III. DAPI/calcofluor co-staining micrographs of cluster III mutants in the presence of formamide for 4 hours at 30°. A wide range of cell cycle defects is observed in this collection of mutants: numbers denote example cells of phenotypes listed in Fig. 3. Scale bar: 10 µm. Micrographs of all remaining mutants at restrictive conditions for four hours are presented in Supplementary Figure 3.

Next, to search for morphology and cell cycle phenotypes, we observed all mutants by DAPI/calcofluor co-staining after four hours in the absence and presence of formamide. Interestingly, we found a wide range of phenotypes: wee-like cells (*fsm*5, 6, 27, 29, 36), elongated cells (*fsm*10, 16, 19, 31, 32, 34, 52), high or low septation index (*fsm*1, 34, 60; *fsm*27), bent cells (*fsm*7, 10, 16, 17, 40, 52), hyper-condensed or relaxed chromatin (*fsm*9, 31, 52, 61; *fsm*1, 7, 34), aberrant nuclear morphology (*fsm*1, 7, 12, 23) and chromosome segregation defects (*fsm*17, 19, 32, 40, 43, 53, 56, 61). The latest include anaphase bridges, *cut* phenotypes^36,37^; bi-nucleated and empty sister cells and cells with a single nucleus but empty sister cell (Fig. 3 right grid). Interestingly, cluster III contains a very comprehensive range of these phenotypes. Thus, these could represent new mutations of interest that have escaped other screenings. Strains in cluster III with remarkable phenotypic differences when grown in the absence and presence of formamide are shown in Fig. 4. The phenotype of all remaining *fsm* strains is shown in Supplementary Figure 3. The phenotypic diversity even within the same cluster also suggests that many *fsm* mutants are unlikely to correspond to the same targeted gene, although alleles with a different penetrance or separation of function cannot be ruled out.

### Formamide specifically targets processes related to RNA metabolism

**Figure 5:**
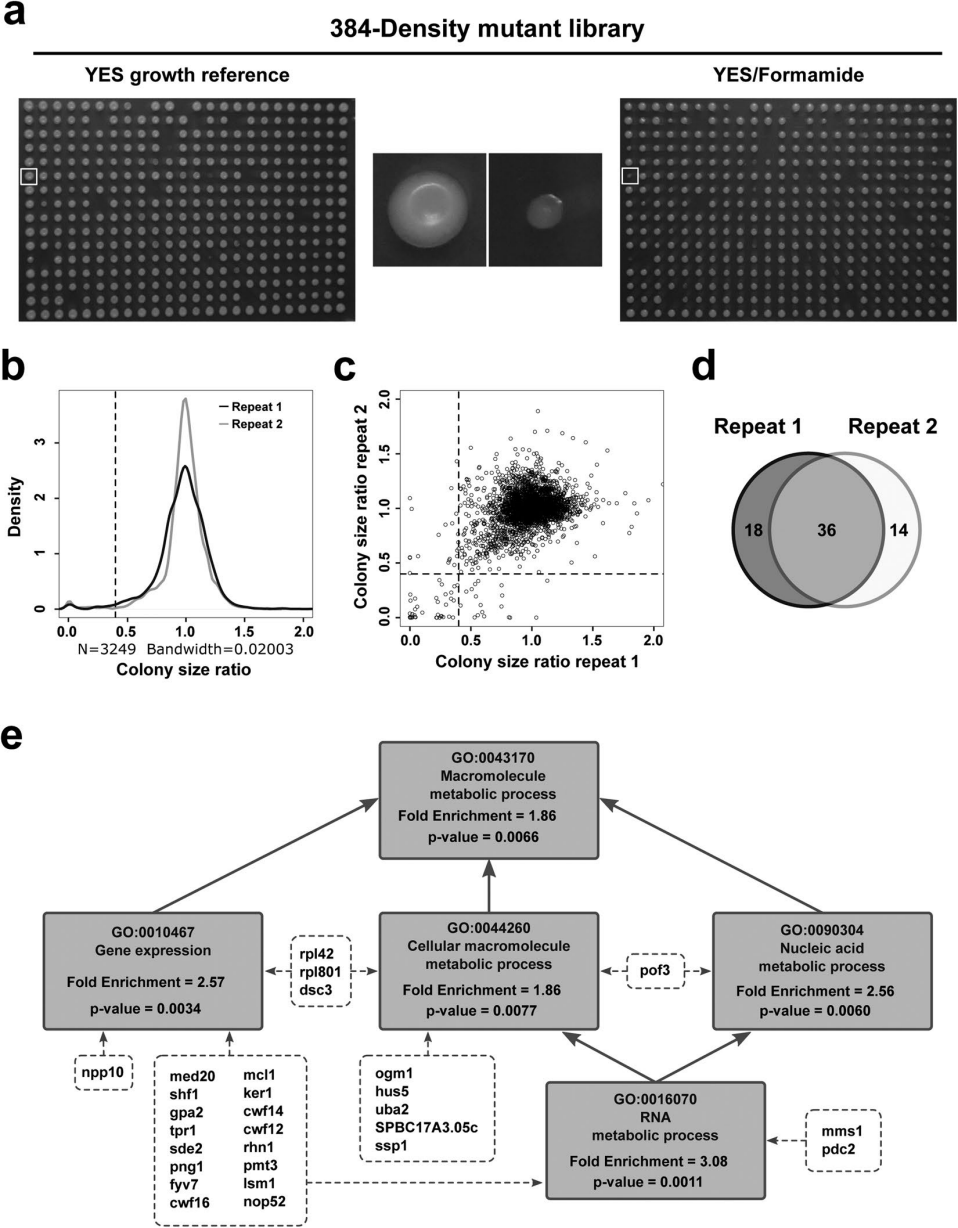
Genome-wide sensitivity screening. (**a**) Non-essential gene deletion collection (Bioneer V5) was arrayed over nine plates in 384-spot density format and copied in YES reference (upper left panel) media as well as in YES-Formamide (2% v/v) (upper right panel) and incubated at 30° for two days. High resolution images were obtained and analysed by computer automated processing to detect defective growth in the presence of formamide. Upper central panels illustrate a random example where a sensitive deletion (white squares) is enlarged in the middle. (**b**) Kernel density plotting for two independent biological repeats of the high-throughput screen. Dotted lines indicate the cut-off used to be considered sensitive. (**c**) Colony size dot plot. Ratio between plate-standardized spot size of each deletion in the two independent biological repeats of the screen (Pearson correlation coefficient = 0.23). Dotted lines represent sensitivity cut-off for both repeats. (**d**) Venn diagram from two biological repeats to identify consistently sensitive candidates. (**e**) Direct hierarchical relationship within the Gene Ontology terms enriched in our study. Boxes include GO term and code, fold enrichment and its associated p-value from AnGeLi analysis. Note that enrichment becomes significant from the most specific (RNA metabolism genes) to the most general GO term (cellular macromolecule metabolic process).

To gain an insight into the biological processes that formamide specifically impairs *in vivo*, we performed a high throughput screen for formamide sensitivity from all non-essential gene deletions in two separate biological repeats. The Bioneer V5 collection^38^ was arrayed in 384 spots format onto standard YES media and incubated at 30° for 3 days. We made copies of each plate on YES, YES-2%formamide and YES-3% formamide media. After three days at 30°, no growth was observed in the presence of 3% v/v formamide, indicating that no gene deletion provides resistance to the drug. We searched for those deletions that were consistently lethal or highly sensitive to 2% formamide in both replicates (Fig. 5a). Images on YES control plates and YES-formamide were obtained and analysed using *Spotsizer* – an automated image analysis platform for colony size measurement^39^. This software standardizes every colony area by the median of its plate and it calculates the ratio between control and experimental condition for each deletion (raw data is provided in Supplementary File 1). In order to remove already sick deletions in control conditions, we filtered out strains with a spot size below 30% of the plate median in YES control plates in both repeats. We then selected deleted loci whose growth was reduced by 60% or more in the presence of formamide in both biological repeats (Fig. 5b and c). Using this stringent cut off, thirty-six loci were identified that, when deleted, lead to high sensitivity to formamide (Table 1, Fig. 5d). The identity of a random sample of 13 of these loci was checked by PCR (Supplementary Figure 4) to confirm gene deletion. Furthermore, these 13 strains were serially diluted and plated serial dilutions on YES and YESF to confirm their sensitivity. Gene deletion was confirmed in 12 out of 13 loci. Sequencing of PCR product from the remaining locus (*cwf16*) determined that the coding region is disrupted by KanMX4 cassette rather than replaced. Nonetheless, ORF interruption at this locus was still predicted to lead to a loss-of-function phenotype. All thirteen selected strains are viable in regular media but unable to grow in the presence of formamide (Supplementary Figure 5).

**Table 1:** Formamide-sensitive deleted loci.

Systematic Gene I.D.	Gene Standard Name	Product	Characterisation Status	Sensitivity score Replicate I	Sensitivity score Replicate II	Human orthologue
SPAC13F5.07c	hpz2	zf PARP type zinc finger protein Hpz2	conserved unknown	0,000	0,000	
**SPAC15E1.03**	**rpl42**	**60S ribosomal protein L36/L42**	**published**	**0,230**	**0,026**	**RPL36AL/RPL36A**
**SPAC17G8.05**	**med20**	**mediator complex subunit Med20**	**published**	**0,038**	**0,022**	**MED20**
**SPAC1F7.13c**	**rpl801**	**60S ribosomal protein L8 (predicted)**	**biological role inferred**	**0,273**	**0,005**	**RPL8**
**SPAC20H4.02**	**dsc3**	**Golgi Dsc E3 ligase complex subunit Dsc3**	**published**	**0,254**	**0,012**	
**SPAC22A12.07c**	**ogm1**	**protein O-mannosyltransferase Ogm1**	**published**	**0,005**	**0,155**	**POMT2**
**SPAC22F8.12c**	**shf1**	**small histone ubiquitination factor Shf1**	**published**	**0,293**	**0,214**	**PPHLN1**
**SPAC23H3.13c**	**gpa2**	**heterotrimeric G protein alpha-2 subunit Gpa2**	**published**	**0,060**	**0,100**	
**SPAC27D7.14c**	**tpr1**	**RNA polymerase II associated Paf1 complex subunit Tpr1**	**published**	**0,102**	**0,046**	**CTR9**
**SPAC30D11.13**	**hus5**	**SUMO conjugating enzyme E2 Hus5**	**published**	**0,242**	**0,343**	**UBE2I**
SPAC31 A2.06	atp25	mitochondrial ATP synthase complex assembly protein Atp25 (predicted)	biological role inferred	0,390	0,296	
**SPAC31G5.18c**	**sde2**	**silencing defective protein Sde2**	**published**	**0,339**	**0,230**	**SDE2**
**SPAC3G9.08ING5**	**png1**	**ING family homolog Png1**	**published**	**0,322**	**0,009**	**ING2/ING3/ING4/**
**SPAC3H8.05c**	**mms1**	**E3 ubiquitin ligase complex subunit Mms1 (predicted)**	**biological role inferred**	**0,005**	**0,000**	
SPAC4F10.06	bud22	ribosome small subunit biogenesis protein, BUD22 family (predicted)	biological role inferred	0,217	0,000	SRFBP1
SPAC869.03c	SPAC869.03c	urea transmembrane transporter (predicted)	biological role inferred	0,262	0,304	
**SPAC8C9.07**	**fyv7**	**rRNA processing protein Fyv7 (predicted)**	**biological role inferred**	**0,000**	**0,000**	**CCDC59**
**SPAC9.13c**	**cwf16**	**splicing factor Cwf16**	**published**	**0,296**	**0,119**	**CCDC94**
**SPAPB1E7.02c**	**mcl1**	**DNA polymerase alpha accessory factor Mcl1**	**published**	**0,243**	**0,000**	**WDHD1**
**SPBC16H5.03c**	**uba2**	**SUMO activating enzyme E1-type Uba2 (predicted)**	**biological role inferred**	**0,337**	**0,173**	**UBA2**
**SPBC1718.03**	**ker1**	**DNA-directed RNA polymerase I complex subunit Ker1**	**published**	**0,042**	**0,006**	
**SPBC17A3.05c**	**SPBC17A3.05c**	**DNAJ/DUF1977 DNAJB12 homolog (predicted)**	**biological role inferred**	**0,019**	**0,001**	**DNAJB14/DNAJB12**
**SPBC19G7.10c**	**pdc2**	**topoisomerase II-associated deadenylation-dependent mRNA-decapping factor Pdc2 (predicted)**	**published**	**0,216**	**0,179**	**PATL1**
**SPBC24C6.11**	**cwf14**	**G10 protein**	**published**	**0,046**	**0,004**	**BUD31**
**SPBC32F12.05c**	**cwf12**	**complexed with Cdc5 protein Cwf12**	**published**	**0,042**	**0,175**	**ISY1**
**SPBC337.03**	**rhn1**	**RNA polymerase II transcription termination factor homolog**	**published**	**0,365**	**0,287**	**RPRD1A/RPRD1B**
**SPBC365.06**	**pmt3**	**SUMO**	**published**	**0,260**	**0,013**	**SUMO1/SUMO1P1**
**SPBC3D6.08c**	**lsm1**	**mRNA decapping complex subunit (predicted)**	**biological role inferred**	**0,221**	**0,000**	**LSM1**
SPBC83.12	SPBC83.12	Schizosaccharomyces pombe specific protein	uncharacterized	0,003	0,211	
**SPBC9B6.07**	**nop52**	**nucleolar protein Nop52 family Rrp1 (predicted)**	**biological role inferred**	**0,359**	**0,157**	**RRP1/RRP1B**
SPBP23A10.16	sdh4	TIM22 inner membrane protein import complex anchor subunit Tim18	published	0,000	0,038	SDHD
**SPCC1739.14**	**npp106**	**nucleoporin Npp106**	**published**	**0,000**	**0,000**	**NUP93**
**SPCC297.03**	**ssp1**	**Ca2+/calmodulin-dependent (CaMMK)-like protein kinase Ssp1**	**published**	**0,228**	**0,144**	**CAMKK1/CAMKK2**
**SPCC338.16**	**pof3**	**F-box protein Pof3**	**published**	**0,353**	**0,001**	**STIP1**
SPCC4B3.10c	ipk1	inositol 1,3,4,5,6-pentakisphosphate (IP5) kinase	published	0,002	0,000	IPPK
SPCC794.12c	mae2	malic enzyme, malate dehydrogenase (oxaloacetate decarboxylating), Mae2	published	0,019	0,033	ME1/ME2/ME3

Non-essential loci deletions that meet specified criteria in both biological repeats to be considered formamide sensitive (see Materials and Methods). 80% of them have at least one manually curated human orthologue in PomBase^42^. “Sensitivity score” corresponds to the spot size ratio between control and experiment plates after normalization. Bold font rows denote genes which belong to significantly enriched GO terms found in this study (indicated in Fig. 5e).

The resulting gene list in Table 1 was analysed by AnGeLi – an online open access software^40^ for gene ontology (GO) enrichment – using standard settings. STRING v10.5 was used for physical and functional interactions. STRING provides an assessment of physical and functional associations within a set of genes and performs statistical enrichment analysis over a given background^41^. AnGeLi results showed a highly significant enrichment in RNA metabolism related genes (p-value = 0.001) and respective hierarchical GOs (Fig. 5e)^42^. Consistently, significant connections (p-value = 0.007) and the same GO enrichment within our gene list were found using the STRING Database (see Materials and Methods and Supplementary Figure 6). These results suggest that RNA secondary structures and/or interactions are especially sensitive to the denaturing properties of formamide inside a living cell.

**Figure 6:**
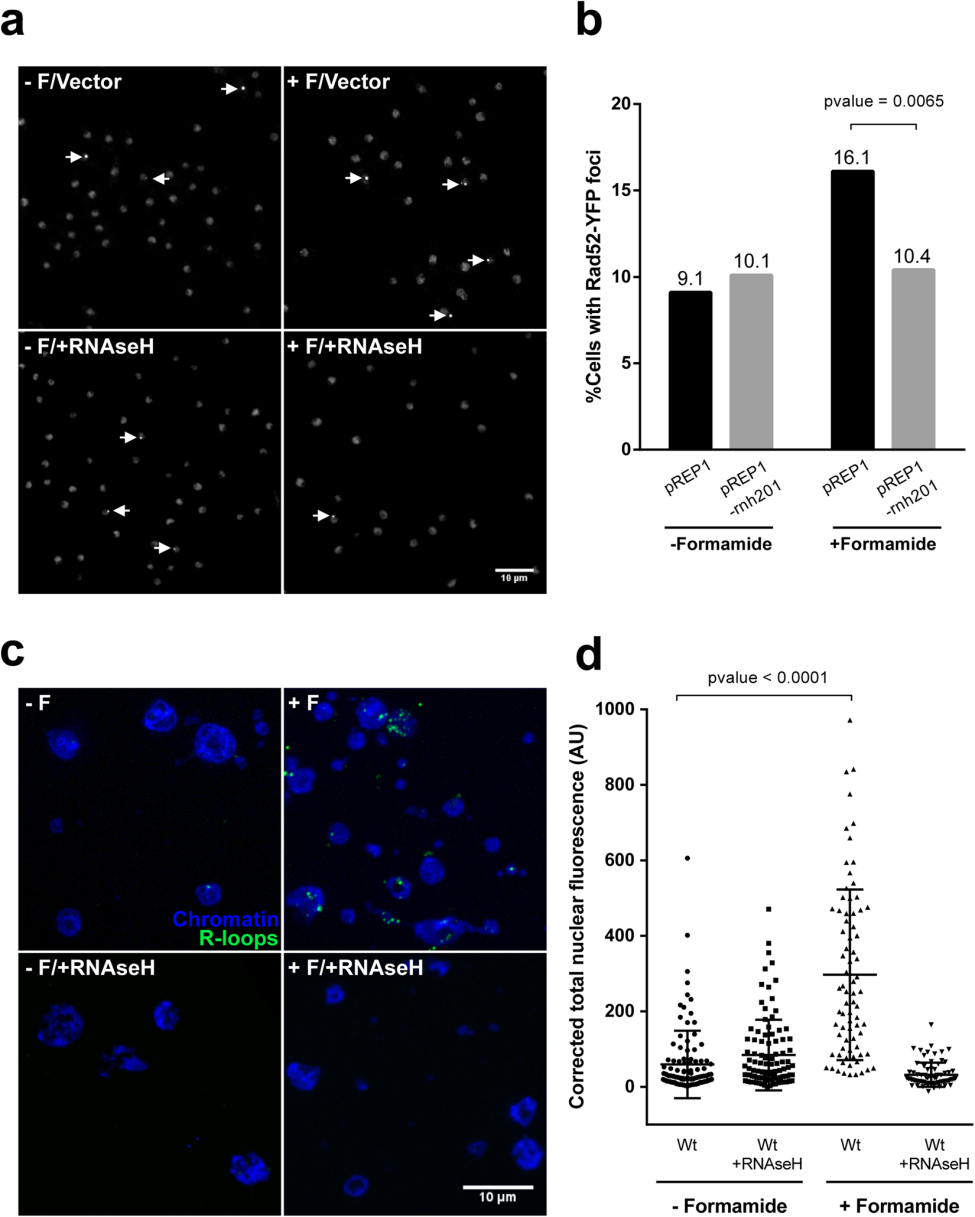
Formamide enhances R-loop formation. (**a**) Examples of average intensity projections of 10 slices stacks from rad52-YFP cells in all four combinations: bearing either pRep1 empty expression vector (upper panels) or over-expressing RNAse H catalytic subunit (Rnh201) under nmt1 promoter (lower panels) and in the absence (left panels) or the presence (right panels) of formamide for three hours. Arrows indicate nuclei containing Rad52 foci. Scale bar: 10 µm. (**b**) Percentage of cells containing Rad52 foci in each condition (n ≥ 520). p-value was determined using a two-sided Chi-square test. (**c**) Chromosome spreads immunofluorescence. Examples of merged DAPI (chromatin)/Alexa488 (R-loops) projections (10 slices stacks) from wild type cells in all four combinations: no formamide no RNAse H (upper left panel), plus formamide no RNAse H (upper right panel), no formamide plus RNAse H (lower left panel), plus formamide plus RNAse H (lower right panel). Scale bar: 10 µm. (**d**) Fluorescent signal quantification in nuclear spreads. Integrated fluorescent density from each nucleus was made relative to the size and field’s average background signal subtracted. Resulting intensity (arbitrary units) is represented for at least 75 nuclei on each condition. p-value was determined using a t-test.

Given this GO enrichment, we next asked if a known downstream phenotype derived from different RNA-related defect could be observed in the presence of formamide in a wild type background. It is well established that a number of alterations in RNA synthesis, processing and transport lead to R-loop formation^43–45^. These structures are formed when RNA molecules associate to complementary DNA strand leaving the other DNA strand exposed to damage^44^. Eukaryotic cells have a number of mechanisms to resolve R-loops^43,44^ including RNAse H activity, which recognizes DNA:RNA hybrids to degrade the RNA strand. Provided that non-resolved R-loops lead to DNA damage, the frequency of these hybrids can be monitored *in vivo* by scoring Rad52 (the repairing recombinase chaperone) foci in wild type and RNAse H overexpression background respectively. If DNA damage is caused by R-loops, the number of cells containing Rad52 foci should be increased (in *S. pombe* this is reported to be about 10%^46^) but reduced back to basal levels when a catalytic RNAse H subunit is overexpressed^47–49^. R-loops can be also detected by using a RNA-DNA hybrid specific antibody (s9.6)^50^ for immunofluorescence on fixed nuclear spreads. Therefore, we asked if formamide could increase the chance of R-loop formation as a readout of RNA biology interference. As can be seen in Fig. 6a and b, a 1.7-fold increase in the number of cells with Rad52 foci is observed in the presence of formamide which drops down to basal levels when the RNAse H catalytic subunit (Rnh201) is overexpressed. RNA-DNA hybrids were also detected in nuclear spreads by s9.6 specific antibody. We observe a significant increase in corrected total nuclear fluorescence (see materials and methods) in formamide-treated cells (t-test p-value < 0,0001). As a specificity control, spreads previously treated with RNAse H showed very low fluorescent signal in any condition (Fig. 6c and d). These observations support the view that the presence of formamide preferentially loosens nucleic acid non-covalent interactions *in vivo* either by hindering double strand DNA re-annealing behind RNA transcription complexes or relaxing RNA secondary structures that in turn increase the chance of RNA-DNA hybrids annealing.

### Formamide diminishes splicing efficiency

**Figure 7:**
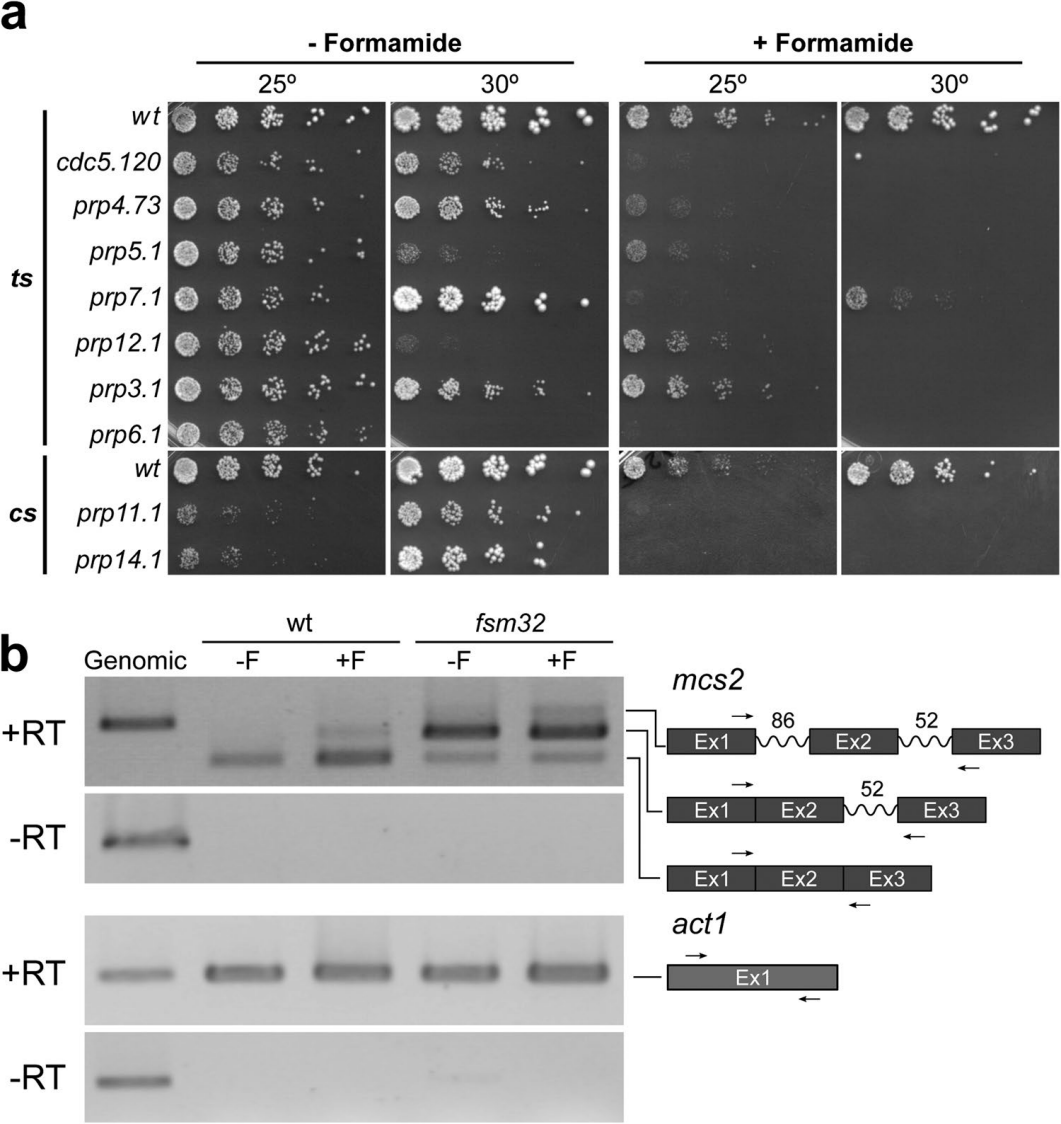
Formamide weakens RNA metabolism. (**a**) Splicing mutants serial spot test dilutions (1/5) grown in the absence (2 left panels) and the presence (2 right panels) at the indicated temperatures on top. Upper panels correspond to reported thermo-sensitive splicing mutants (ts) and lower panels to reported cold-sensitive splicing mutants (cs). All mutants die in the presence of formamide even at their permissive or semi-permissive temperature. (**b**) rtPCR analysis from *mcs2* intron-containing gene. Total RNA was extracted from wild type and *fsm32* mutant cells grown in the absence and presence of formamide for 6 hours. After DNAse treatment half of the sample was subjected to reverse transcription (upper panel) using random primers and the other half was used as a control to assess digestion of residual genomic DNA (middle panel). PCR amplification was performed using intron-flanking primers as indicated in the chart on the right. Numbers denote intron length in base pairs. Spliced and non-spliced forms can be distinguished by band size. Actin was used to standardize samples (lower panels).

A mutant displaying clear cell cycle defects at the restrictive condition (*fsm 32*) was chosen for further characterization. To identify the mutated gene, we rescued the lethality by complementation with a genomic library^51^. Sequencing of cloned genomic fragments and linkage analysis identified the locus SPBC337.06c as the wild type version of *fsm*32. Mutant *fsm32* is allelic to *cwf15* (Complexed with Cdc five), an essential and evolutionary conserved gene, predicted to be a splicing factor^52^. Given that a mutant allele of a spliceosome factor (*cwf15/fsm32*) as well as three other *cwf* gene knock-outs (*cwf12∆*, *cwf14∆* and *cwf16∆*) identified in this study, cause a severe lethality in the presence of formamide, we hypothesized that mutations in critical elements of RNA metabolism such as the splicing machinery, should lead to marked formamide sensitivity. To test this hypothesis, we plated a collection of 9 splicing mutants in the absence and presence of formamide at different temperatures. These mutants had been previously isolated as either thermo or cold-sensitive strains in different genetic screenings (Supplementary Table 1)^53–57^. As shown in Fig. 7a, all tested mutants were unable to grow in the presence of formamide even at their permissive temperature. Conversely, we determined whether sub-lethal concentrations of formamide can diminish the splicing efficiency both in the wild type and *cwf15.32* background. We analysed splicing intermediates by rtPCR in the presence and absence of formamide from the intron-containing gene *mcs2*.This locus has previously been used as a defective splicing reporter^58^. As shown in Fig. 7b, whilst only the processed band is detected in regular growth condition, intron-containing mRNA molecules are detected in the presence of formamide in the wild type. We also observe a clear splicing defect in *cwf15.32* mutant in both conditions. Although this is exacerbated in the presence of formamide, this mutation is leaky at the permissive condition.

## Discussion

In recent years, following the discovery of different types of RNA molecules, many fascinating regulatory functions have been identified in virtually all biological processes^59^. Apart from the intrinsic information carried by messenger molecules, RNA spatial structure is absolutely critical for these functions, many of which mostly depend on intra and inter-molecular interactions. Furthermore, disruption of the spatial interplay within and between RNA molecules, or between RNA and protein underlies many human diseases^60^. Because of this, our ability to study these interactions in model organisms may prove informative in our understanding of the molecular mechanisms of disease and the development of new treatments. Here using fission yeast, we show that formamide preferentially targets *in vivo* RNA biology over any other kind of biomolecular function and that it can be used as a selective or synergistic system to diminish RNA intra and intermolecular interactions., It may therefore represent a valuable tool to probe various aspects of RNA biology, such as biosynthesis, processing, and functional interactions.

As opposed to budding yeast, which can cope with 3% v/v, here we show that fission yeast cells are only able to grow up to saturation in the presence of ~2% v/v formamide. Nevertheless, the goodness of fit to a logarithmic growth line decreases as concentration rises. This effect could be due to the denaturing properties of this solvent (see below). We wanted to know whether this might provide a useful synergistic selective condition to isolate mutations that loosen tertiary structures, and/or molecular interactions which become fully impaired in the presence of this chemical, leading to a loss of a specific biological function. In line with this view, we found 72 of these mutants and further analysed 35 of them, which have been named *fsm* (formamide sensitive mutant). These strains showed very heterogeneous co-sensitivities with other widespread restrictive conditions used in fission yeast. By clustering formamide sensitive mutants according to their sensitivity to eight different conditions, we gathered three clusters: I) Sensitive to most (2–7) conditions, II) sensitive to just one extra condition and III) only sensitive to formamide. We show that in cluster I and II sensitivity to formamide co-segregates with other sensitivities suggesting that, in these cases, the single mutation effects are pleiotropic. An example of these is *fsm32* (cluster I), which was isolated and identified in this study. It corresponds to a novel mutant allele on *cwf15*, a very well conserved but non-characterized spliceosome component^61,62^. Cloning of this mutant by complementation also shows that formamide sensitive mutations can be rescued by ectopic expression of their respective wild type alleles, or multicopy extragenic suppressors. A preliminary observation by DAPI/calcofluor co-staining in the presence of formamide revealed a wide range of cell cycle progression defects in a significant proportion of strains (28 out of 35), with chromatin segregation defects particularly prominent. Cluster III mutants are not sensitive to six other extensively used drugs, or low or high temperature. Although other conditions might be considered, formamide´s biological mode of action does not fully overlap with any of the tested conditions. We found 11 out of these 13 mutants display penetrant cell cycle phenotypes which could bear mutations in uncharacterized genes, - or new alleles of already known genes - involved in cell cycle progression, which could have escaped other searches. It will be interesting to characterize these mutants further and eventually identify the target loci as these could represent novel connections between RNA molecules/Ribonucleoproteins and critical steps in cell cycle progression.

With the aim of identifying potential targets of formamide, we assessed the sensitivity of non-essential deletion mutants in a genome-wide screen. We found 36 deletions that cause formamide to become lethal to cells, or diminish growth by ≥60%. Statistical analysis showed that 50% of the corresponding genes fall under “RNA metabolic process” and “gene expression” Gene Ontology classifications and 72% are physically or functionally connected. Such a significant enrichment strongly suggests that RNA related functions are especially sensitive to formamide *in vivo*. From the description by Aguilera (1994) of formamide sensitivity as a novel conditional phenotype in yeast, to our knowledge, only ten formamide-sensitive alleles of known genes in *S. cerevisiae* and five in *S. pombe* have previously been identified in different studies. In agreement with our data, it is important to note that twelve out of fifteen of these genes have been directly related to RNA biogenesis or processing: pre-mRNA cleavage and polyadenylation factor YTH1^28^, transcription-related HTZ1 (histone H2A.Z)^29,63^, members of the RSC complex in *S. cerevisiae* and *S. pombe* - RSC8/SWH3, Arp9, Arp42, and Rsc1 -, that remodel chromatin and play an important role in transcription elongation^30,31,64^. ACT3/ARP4, Spt6 and Iws1 that have been also shown to have transcription elongation activity^32,33^. NOP2, which works in the processing of 27S pre-rRNA into mature 25S rRNA^34^. RTR1 (regulator of transcription) encodes an RNA pol II CTD phosphatase^35,65^. One other *fs* mutant allele was also found to function in conjunction with nucleic acids: CDC9, a DNA ligase essential for Okazaki fragments joining and ribonucleotide excision repair^22,66,67^. Out of fifteen genes, only CAN1 (arginine permease), CDC42 GTPase and YIP1 (COPII vesicle biogenesis^68^) have not been directly related to RNA metabolism. Thus, consistently with completely independent previous studies, we find that the majority of gene products identified as targets of formamide *in vivo* are associated with RNA biogenesis and metabolism. This said, although there was no significant GO enrichment, a handful of other non-RNA related targets were also identified in our high-throughput screen such as transporters and metabolic enzymes.

The fact that some deletions identified in our screen correspond to splicing factors such as *cwf12*, *cwf14* and *cwf16*, and that one mutation identified in this study *(fsm32)* is allelic to *cwf15*, an essential predicted spliceosome component, prompted us to ask if a central step in RNA maturation such as splicing is compromised in the presence of formamide. In agreement with this view, we show that a collection of previously isolated mutations in splicing factors, become severely sensitive to formamide at otherwise perfectly viable conditions. Although formamide effects on protein structure in mutant alleles cannot be ruled out, it is possible that the critical structural relationship between ribonucleoproteins and their target RNA molecules in these cases is uncoupled by formamide at permissive temperature by synergistic effects of a leaky protein recognition motif and loose RNA secondary structure. Furthermore, in the presence of the drug, intron-containing mRNA molecules are detected by rtPCR not only in *fsm32/cwf15* mutant but also in the wild type background; suggesting that formamide negatively affects the splicing efficiency *in vivo*.

R-loops were described four decades ago as *in vitro* structures in particular conditions where RNA was able to hybridize to a complementary DNA strand, displacing the antiparallel strand^69^. Later on, it was discovered that transient R-loop formation *in vivo* can serve for regulatory function^70–72^ but R-loops also form as a consequence of defective RNA synthesis, processing or transport. Stabilization and/or accumulation of R-loops can be highly deleterious for the cell^49,73,74^. Here we show that formamide’s presence in the media at a concentration of 2% leads to a significant increase of this specific RNA-related phenotype in living cells and fixed nuclei. R-loops could be formed either by relaxing intramolecular RNA secondary structure interactions or by disrupting inter-molecular RNA-proteins interactions. These weak interactions could be affected by low concentrations of formamide, leading to the unfolding of nascent RNA, possibly leaving it nude, thus making it more prone to anneal to complementary DNA. Once formed, RNA:DNA hybrids have even greater thermodynamic stability than their DNA-DNA counterparts^69^. This would explain why low concentrations of formamide would favour formation of R-loops but not their removal. However, as we show, this excess of R-loops can be suppressed by increasing RNAse H enzymatic activity in the presence of formamide. Although non-lethal at the concentration used, the R-loop increase we observe could account for at least part of the duplication time delay observed in Fig. 1b.

Taken together, the observations discussed above lead us to propose that functions involving RNA molecules are more prone to be affected by formamide *in vivo* before any other biomolecule. In applied terms formamide may serve as a stringent selection method to enrich for RNA related mutants. As shown with splicing mutants, it could also be considered as an alternative switch for already known ribonucleoprotein mutants to avoid the stress-associated effects of temperature shifting. This can be especially useful when working with double mutant backgrounds where two different switches are required, or to increase synergism with other hypomorphic mutations. This chemical could also be an alternative in model organisms where temperature homeostasis is very strict. Although out of the scope of this work, assuming that enhanced R-loop hybrids arise from its RNA denaturing properties, it could be interesting to test if adding low amounts of formamide to *in vivo* RNAi experiments results in more effective hybridization - as it does in FISH protocols for fixed cells - thus enhancing target depletion.

## Methods

### Media and growth conditions

Standard fission yeast growth media and molecular biology approaches were used throughout^75^. For spot tests, cells were grown to midlog phase, cell/ml was scored and matched for all cultures. Serial fivefold dilutions were plated onto solid media. Indicated v/v percentage of formamide (Sigma F7503) was added to solid or liquid media for sensitivity assays. Where it is not specified 2% v/v was used. Phloxine B (Sigma P2759) was used at 2,5 mg/ml to assist the identification of *fsm* strains. Sporulation agar (SPA) was used for mating and sporulation. Singer MSM 400 automated dissection microscope (Singer Instruments) was used for tetrad pulling.

### Isolation of formamide sensitive mutants

A h+ ura4.D18 leu1.32 ade6.M216 strain was mutagenized using either ultraviolet light, N-ethyl-N-nitrosourea (ENU; Sigma N3385), ethyl methanesulfonate (EMS; Sigma M0880) or methyl methanesulfonate (MMS; Sigma 129925)^76^. Cells were grown overnight to mid log phase in 150 ml YES medium at 30° in all cases. For chemical mutagenesis cells were concentrated to 2·10^8^ cells/ml and 1 ml aliquots were washed twice with 1 ml of water, resuspended into 1.5 ml of 50 mM potassium phosphate or 0.1 M sodium phosphate buffer (pH 7.0) and split into two. One half of each was used as a control to estimate cell survival and the other contained either 1% MMS, 0.3 M EMS or 0.05 M ENU respectively. Different aliquots were incubated 30 minutes with agitation at 30 °C and inactivated by adding 1.3 ml sodium thiosulfate (10%), 8 ml sodium thiosulfate (5%) respectively for 10 minutes or washing up cells with media three times in the case of ENU. Cells were then washed twice with YES and plated onto YES media (500 cells/plate). For UV mutagenesis, we took an aliquot of 2 ml from the log phase culture. Cells were harvested and resuspended in a final volume of 1 ml of media (1,4·10^8^ cells/ml). Cells were plated on YES media at the concentration of 500 cells per plate, and exposed to 50 J/m^3^. Three control plates were not irradiated to assess cell survival. After mutagenesis, in all cases plates were incubated 4 days at 30°, and then replica-plated onto YES and YESFPh (YES 2% formamide and 10 mg/L Phloxine B). Colonies unable to grow in the presence of 2% formamide v/v were isolated and double checked in YESF fresh plates.

### Qualitative spot test assay

Every *fsm* was inoculated from a plate in a unique well of a 96-well plate. After 13 hours, they were diluted 10-fold and plated onto YES and YES containing the following drugs: calcofluor white, 0.58 mg/ml; formamide, 2% v/v; camptothecin, 23 μg/ml; cycloheximide, 12.5 μg/ml; hydroxyurea, 0.65 mg/ml; and methyl methanesulfonate, 0.01%. Plates were incubated for five days at 20°, four days at 30° and three days at 36°. Plates were manually analysed in order to assess the sensitivity of every strain by comparing the growth on rich YES media against all other conditions. Colours were used to represent: “non-sensitive” (white), “slow growers” (grey) and “highly sensitive or lethal” (black), respectively in comparison with wt controls on each plate. Strains were grouped in clusters depending on the number of conditions different from formamide that mutants were sensitive to: Two or more (cluster I), one (cluster II), only formamide (cluster III).

### Microscopy

For DAPI/calcofluor staining, cells were grown to early log phase. After 4 hours of 2% v/v formamide incubation, cells were fixed adding 900 μl of culture to 100 μl formaldehyde (37%). After 15 min of incubation on ice, cells were washed 3 times with cold 1x PBS. A small fraction of cells was extended onto a coverslip and allowed to dry out. Then, cells were stained with 5 μl of mounting solution (38 μl glycerol 50%, 46.5 H2O, 10 μl Antifade (p-phenylenediamine, Sigma P6001, 10 mg/ml in phosphate-buffered saline pH 8.2), 3 μl DAPI (4′, 6-diamidino-2-phenylindole, Sigma D1388, 0.1 mg/ml) and 2.5 μl calcofluor white (Sigma F6259, 0.35 mg/ml in water)^77^. Images were taken in a Nikon ECLIPSE Ti-S microscope with a Plan Apo VC 60x/1.40 Oil NA lens. For immufluorescence, protoplasts were prepared as described in Flor-Parra *et al*.^78^ and chromosome spreads according to Loidl and Lorenz^79^ with the only exception of using 1% NP40 detergent (USB 19628) instead of lipsol. Slides were incubated with s9.6 antibody (Sigma MABE1095) diluted 1:2000 in blocking buffer. Anti-mouse IgG Alexa488-conjugated (Invitrogen A11029) was used as secondary antibody diluted 1:2000. Control samples were treated with RNAse H (Roche 10786357001) at 3 u/100 μl for two hours. Nuclei were stained with DAPI. Presented nuclear spreads and Rad52-YFP images correspond to average intensity projections of 10 images stacks with a Z-step of 0.3 microns obtained in a spinning disk confocal microscope (Yokogawa CSU-X1 head mounted on Olympus body; CoolSnap HQ2 camera (Roper Scientific), Plan Apochromat 100X, 1.4 NA objective (Olympus)). Rad52-YFP cells were immobilized with soybean lectin (Sigma L1395) in glass-bottom microwell dishes (MatTek P35G-1.5-10-C). Image analyses were performed using Image J open software^80^.

### rtPCR

Total RNA was extracted as in Rougemaille *et al*.^81^. 10 μg were treated with DNAse (NEB M0303S) and subjected to reverse transcription by random primers using high capacity cDNA Reverse Transcription kit (Applied Biosystems 4368814) following manufacturer instructions. PCR was performed from resulting cDNA using respective intron flanking primer couples listed in Supplementary Table 2.

### Cloning of *fsm32* mutant

A mutant displaying clear cell cycle defects at the restrictive condition (*fsm* 32) was chosen for further characterization. First, we cleaned it up genetically by crossing three times to a wild type background, checked for monogenic segregation (Supplementary Figure 1) and recessiveness in stable diploids. To identify the mutated gene, we rescued the lethality by complementation. The selected mutant strain was transformed with pURB2 based genomic library^51^, recovered in EMM without uracil for four hours and plated directly onto 2% formamide containing media. An aliquot was plated onto a plasmid-selective media with no formamide to estimate transformation efficiency. A total number of 85000 transformants were obtained. Plasmids were recovered out of the candidates grown at restrictive conditions and amplified in *E*. *coli* (DH5α). We then classified clones by restriction analysis into 3 groups. A representative of each restriction group was re-transformed into the mutant strain to assure plasmid–mediated complementation. Only one resulted in clear complementation ability which was sequenced to identify complementing genomic region. Linkage analysis confirmed mutated locus.

### Formamide sensitivity screening

A genome-wide set of gene deletions^38^; (Bioneer Version 5) was arrayed in 384 spot format in YES+ G418 (100 mg/L) and copied onto YES, YES/2% and YES/3% formamide plates respectively by using Rotor HDA robot (Singer Instruments). Two biological repetitions were performed. It is to note that, setting up the conditions of this assay, we found much clearer growth difference of sensitive strains between YES and YESF by pinning sterile water on top of the cell patches previously deposited. This creates a uniform layer of cells in each spot and it probably also resuspend nearby drug making it more accessible and homogeneous within the cells patch. Image automated analysis (see bioinformatics tools) standardized size of each colony by the median of its plate. We considered very sick, and excluded from further analysis, deleted loci that exhibit, in both biological repetitions, a normalization equal or below 0.3 against the median of its plate in regular YES media. For all remaining strains above this threshold we calculated the ratio between normalized colony size on YES and YES/formamide (Supplementary File 1). Deletions that reduced growth by 60% or more (ratio below 0.4) in the presence of formamide in both biological repeats were considered as formamide sensitive deletions.

### Bioinformatics tools

Automated colony size ratio for genome-wide formamide sensitivity screen was performed using *Spotsizer* open software(ref.^39^; http://doi.org/10.4225/08/57315CD446E2C). GO enrichment analysis was performed by online *AnGeLi* software(ref.^40^; www.bahlerlab.info/AnGeLi) using standard parameters. Physical and functional protein associations were obtained by using STRING database version 10.5(ref.^41^ https://string-db.org/). The minimum required interaction score was set to 0.150. Version 5 (Bioneer) deletion collection list was used as the reference background for AnGeLi and STRING analysis.

### Loci deletion verification

We checked 13 random formamide sensitive deletions obtained in the screening from Bioneer V5 collection (*cwf14, cwf16, fyv7, gpa2, ker1, lsm1, mcl1*, *med20*, *nop52*, *png1*, *pof3*, *rhn1*, *tpr1*) by 3 different PCR reactions from each genomic DNA and a wild type control strain for each case. Primers used are listed in Supplementary Table 2.

### Data Availability

All data generated or analysed during this study are included in this published article and its Supplementary Information files.

## Electronic supplementary material

Supplementary Information

File1
