# Supplementary material for: RNA metabolism is the primary target of formamide in vivo
Source: Sci Rep. 2017 Nov 21;7:15895. doi: 10.1038/s41598-017-16291-8 (PMC5698326; doi:10.1038/s41598-017-16291-8)
Supplement: Supplementary file 1 — Supplementary Information [file 41598_2017_16291_MOESM1_ESM.pdf]

Supplementary Information for

RNA metabolism is the primary target of formamide *in vivo*

Rafael Hoyos-Manchado[1§], Félix Reyes-Martín[1§], Charalampos Rallis[2,3], Enrique Gamero-Estévez[1,4], Pablo Rodríguez-Gómez[1,5], Juan Quintero-Blanco[1], Jürg Bähler[2], Juan Jiménez[1] and Víctor A. Tallada[1]*

[1]Centro Andaluz de Biología del Desarrollo, Universidad Pablo de Olavide/Consejo Superior de Investigaciones Científicas, Carretera de Utrera Km1, 41013 Seville, Spain. [2]Research Department of Genetics, Evolution and Environment and UCL Institute of Healthy Ageing, University College London, WC1E 6BT London, United Kingdom. [3]School of Health, Sport and Biosciences, University of East London, E15 4LZ London, United Kingdom. [4]Department of Human Genetics, McGill University, Montreal, H3A 0C7 Quebec, Canada. [5]Human Brain Mapping Unit, Instituto Pluridisciplinar, Universidad Complutense de Madrid, 28040 Madrid, Spain.

§ These authors contributed equally to this work.

Correspondence to:

Víctor A. Tallada: valvtal@upo.es

Supplementary Figure 1

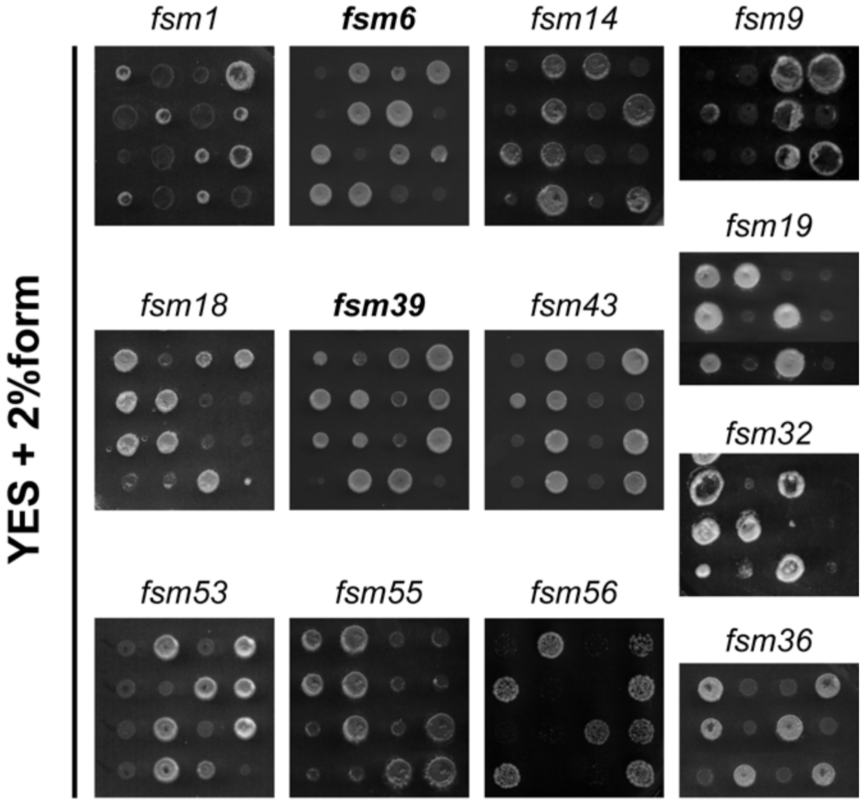

Genetic segregation of formamide sensitivity causing mutations. Thirteen *fsm* mutants were crossed to a wild type. Resulting tetrads for each mutant were replica-plated to formamide to assess monogenic segregation. Only two of them (*fsm6* and *fsm39*) did not show the expected 2:2 ratio for a single gene mutation.

Supplementary Figure 2

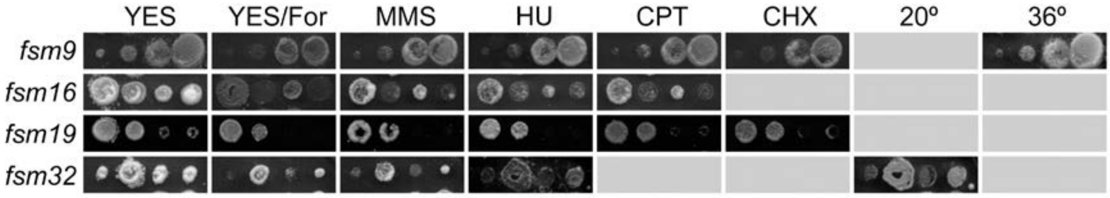

Multiple drug sensitivities linkage to *fsm* loci. Tetrads from cluster I monogenic mutants assessed in Supplementary Figure 1. were replica-plated to other particular restrictive conditions to monitor corresponding co-segregation. One tetrad example from each mutant tested is shown in respective conditions. Empty boxes correspond to non-sensitive conditions for each mutant.

Supplementary Figure 3

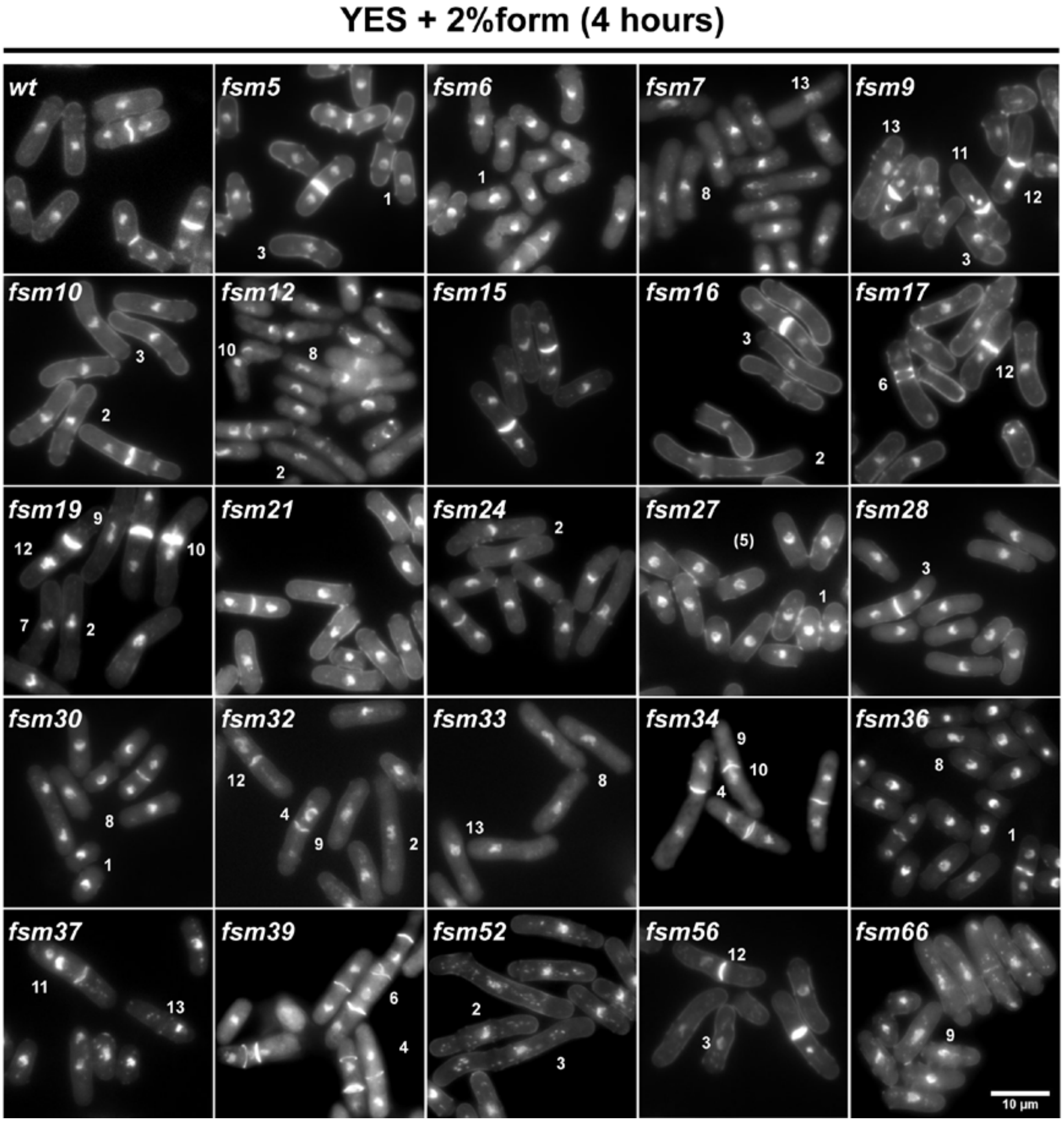

Phenotype range in *fsm* strains in Clusters I and II. DAPI/calcofluor co-staining micrographs in the presence of formamide of all mutants not shown in Figure 4. Numbers denote example cells of phenotypes listed in Figure 3. Scale bar: 10µm.

Supplementary Figure 4

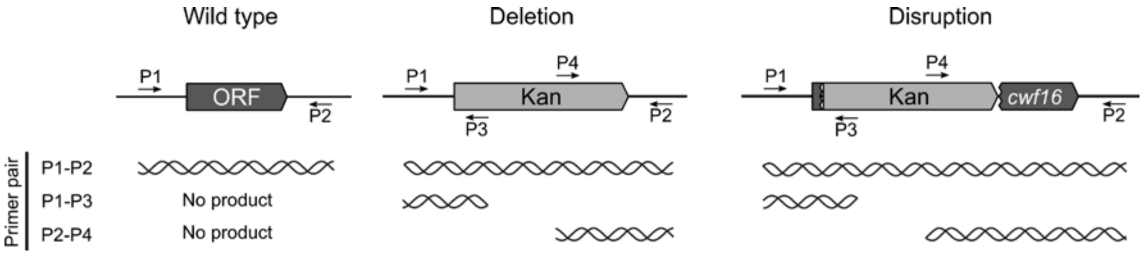

PCR verification of individual deletions identified in the genome-wide screening. Schematics of PCR strategy to verify individual ORF deletions.

Supplementary Figure 5

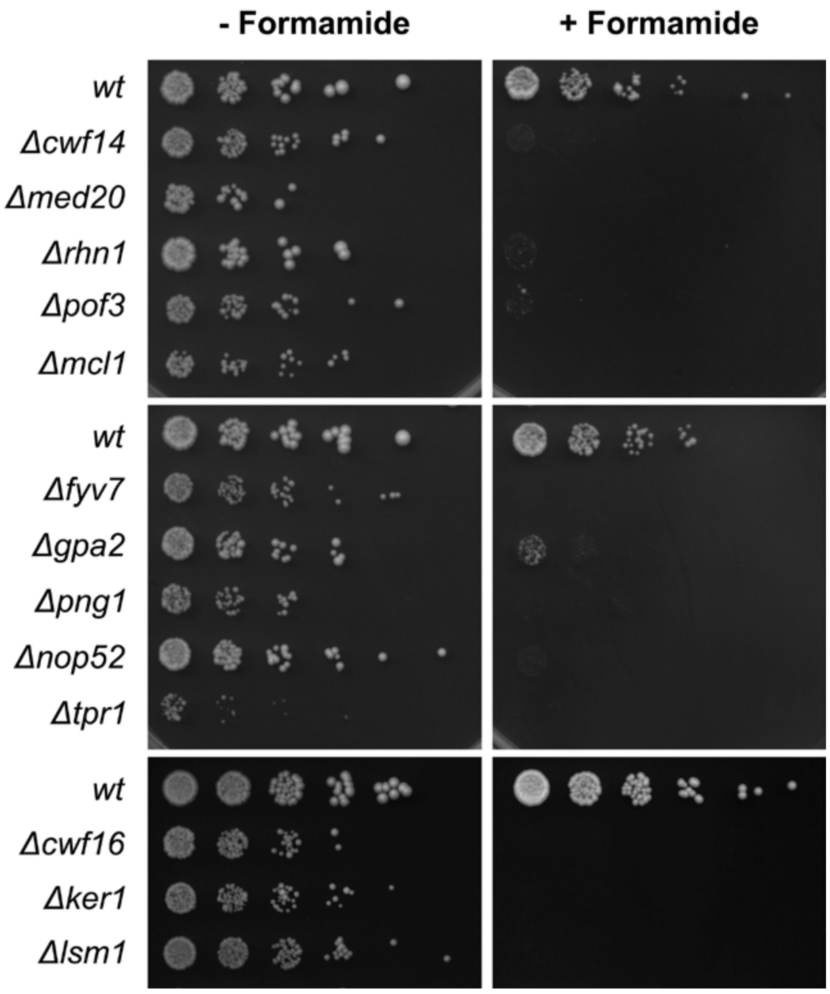

Formamide sensitivity verification of individual deletions identified in the genome-wide screening. Serial spot test dilutions (1/5) in the absence (left) and the presence (right) of formamide of a representative number of sensitive candidate deletions (PCR-verified) identified in our high throughput screening.

Supplementary Figure 6

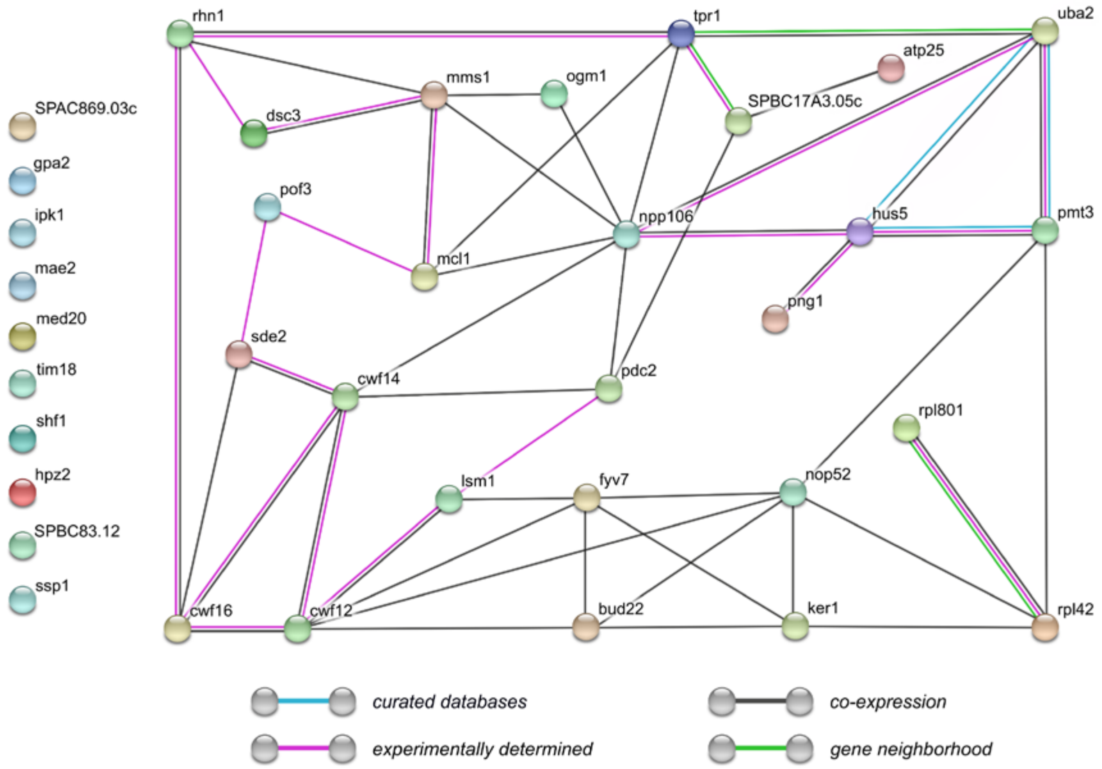

Associations network within gene product corresponding loci identified in the genome-wide screening. A network showing the protein-protein associations for the 36 formamide sensitive deletions was drawn using STRING (version 10.5, {Szklarczyk, 2017 #235}). Interactions supported by experimental data, curated databases, neighbourhood and co-expression were included. In order to perform an enrichment analysis, the Bioneer deletion collection was established as statistical background, excluding the sick deletions that were filtered out in the previously described high throughput screening. Our protein set displays more interactions (49 edges) among themselves than what would be expected (34 edges) for a random set of proteins of same size taken from the reference background (PPI enrichment p-value = 0.0073). This analysis shows that this set was also enriched in two GO Biological Process terms: GO:0010467 (gene expression) and GO:0016070 (RNA metabolic process), as it was pointed out by AnGeLi algorithm.

Supplementary Table 1

Systematic ID	Allele	ts or cs	GO Biological Process	Human ortholog	Reference
SPAC29E6.02	prp3	ts	mRNA cis splicing, via spliceosome	PRPF3 (pre-mRNA processing factor 3)	Potashkin et al (1989): Pre-mRNA splicing mutants of Schizosaccharomyces pombe
SPAPJ698.03c	prp12-1	ts	mRNA cis splicing, via spliceosome	SF3B3 splicing factor 3b subunit 3	Urushiyama et al (1996): Isolation of novel pre-mRNA splicing mutants of Schizosaccharomyces pombe
SPCC777.14	prp4-73	ts	mRNA cis splicing, via spliceosome, negative regulation of G0 to G1 transition, protein phosphorylation	PRPF4B pre-mRNA processing factor 4B	Rosenberg et al (1991): prp4 from Schizosaccharomyces pombe, a mutant deficient in pre-mRNA splicing isolated using genes containing artificial introns
SPBP22H7.07	prp5-1	ts	mRNA cis splicing, via spliceosome	PLRG1 pleiotropic regulator 1	Potashkin et al (1998): Cell-division-cycle defects associated with fission yeast pre-mRNA splicing mutants
SPCC10H11.01	prp11-1	cs	mRNA cis splicing, via spliceosome, U2-type prespliceosome assembly	DDX46 DEAD-box helicase 46	Urushiyama et al (1996): Isolation of novel pre-mRNA splicing mutants of Schizosaccharomyces pombe
SPAC644.12	cdc5-120	ts	splicing factor, Prp19 complex subunit Cdc5	CDC5L	Nurse et al (1976): Genetic Control of the Cell Division Cycle in the Fission Yeast Schizosaccharomyces pombe
	prp6-1	ts			Potashkin et al (1998): Cell-division-cycle defects associated with fission yeast pre-mRNA splicing mutants
	prp7-1	ts			Potashkin et al (1998): Cell-division-cycle defects associated with fission yeast pre-mRNA splicing mutants
	prp14	cs			Urushiyama et al (1996): Isolation of novel pre-mRNA splicing mutants of Schizosaccharomyces pombe

Splicing mutants tested in this study. Seven temperature-sensitive (ts) and two cold-sensitive (cs) splicing defective mutants previously isolated were used to test sensitivity to formamide under their permissive temperature. The ones whose product has been identified have all a human orthologue as indicated.

Supplementary Table 2

Primer	Sequence
Bioneer CPC1	TGATTTTGATGACGAGCGTAAT
Bioneer CPN1	CGTCTGTGAGGGGAGCGTTT
cwf12 F	TGAAAGGGACCATTTATGCAATAAT
cwf12 R	AAAAAGTCGCCCTATATAAAAACGA
cwf14 F	CATCTGGTTCCCTTTTGGCC
cwf14 R	TCAGCAACAAATGATGGACC
cwf16 F	AAAGTACTCTTAAAACACCGGAAGG
cwf16 R	AGACGTTCCTATCATAGTTCC
fyv7 F	CCGAGTAATGGCTCCATAACAAATT
fyv7 R	ATCTTGCGTTGTGGTTTTTTCTCAG
gpa2 F	CCCATAAAGGGGGTTAATCAAACTT
gpa2 R	TTCAATAACTTTAAAAGCATTCGCC
ker1 F	TGCTTACGACATTATAAATAGGCGG
ker1 R	TTGAGTTTAGTAATTGATCTCGCCA
lsm1 F	GAGCCTTTCAATGAACTAATCCGC
lsm1 R	ATTACGGATGTGGGCAATGGG
mcl1 F	TAGCTAATTGCATATTTTTCCTCCA
mcl1 R	GTTGTACTGCACTTAGTGTCTGCAA
med20 F	GAGACACATATGTAACATCCTGGG
med20 R	CCTCCTTTGAAGTACCAACAAGG
mms1 F	ATTTTAAATGGCCGTAGAAAATTCA
mms1 R	AACCTGGATTCGCCAGAAACATTAT
nop52 F	TCTCCTTTTCAAAGGCATGC
nop52 R	GTGGTCGTGGGTTCGATTCC
pdc2 F	TGTAGCTTGCTTGAGCAAGC
pdc2 R	CGTCGACGTGTCAGATTGGG
png1 F	GGGCTACTATAAACAACCGG
png1 R	CTACGGCGTGTAATATCTGC
pof3 F	GCTTCAGTGCAAAATAGTAAACACC
pof3 R	CTCGTTTTAGCGACGAAGGATGTTA
rhn1 F	GGGAATCTTGGAGAAAACTGGG
rhn1 R	CAGATATATCAGTCTCAGACGG
sde2 F	CTGCTTTCCAATAGTCGTATAAGGG
sde2 R	TGATAGTCCATTGTGTTTTGAATGC
tpr1 F	AGCAGCACATTCCGACACCCTC
tpr1 R	CGTTTCCCTTTTACGCTTCCATC
mcs2 Ex1 F	GCACTTTCTTCCGCTCTTTCC
mcs2 Ex3 R	TTTCGGAAGCACTGTTTGACAATC
act1 F	CCCCTAGAGCTGTATTCCC
act1 R	CCAGTGGTACGACCAGAGG

Deletion checking Primers. List of primers used in this study for respective deletion check-up in corresponding strains in Supplementary Figure 4 and primers used for rtPCR in figure 7b.

Supplementary File 1

SupplementaryFile1.xlsx

Whole deletion library response to formamide crude data. Median-standardized values by *Spotsizer* software for Control and Experiment conditions as well as ratio for each spot are listed for both biological repeats, labelled as “Exp1” and “Exp2” respectively.
